# Healthcare workers hydration status and performance: a scoping review

**DOI:** 10.3389/frhs.2026.1778164

**Published:** 2026-08-25

**Authors:** Omar B. Da’ar, Razan Alhabeeb, Farah Kalmey

**Affiliations:** 1 Health Systems Management Department, College of Public Health and Health Informatics, King Saud bin Abdulaziz University for Health Sciences, Riyadh, Saudi Arabia; 2 King Abdullah International Medical Research Centre, Riyadh, Saudi Arabia; 3 Ministry of National Guard, Riyadh, Saudi Arabia; 4 Ministry of Health, Riyadh, Saudi Arabia; 5 College of Science and Health Professions, King Saud bin Abdulaziz University for Health Sciences, Riyadh, Saudi Arabia

**Keywords:** healthcare workers, hydration, performance at work, Saudi arabia, well-being

## Abstract

**Background:**

Healthcare workers play a crucial role in providing life-saving care in high-pressure fast paced settings, where sustained alertness is critical. Adequate hydration is essential for their decision-making and timely delivery of treatment. Water intake is crucial for optimal cognitive, digestive, and cardiovascular function. The World Health Organization (WHO) emphasizes the importance of hydration in delivering quality healthcare. Therefore, this review aims to map the existing literature on healthcare workers’ hydration status and its relationship with their work performance.

**Methods:**

In this scoping review, we conducted an electronic search in three databases- PubMed/PubMed Central, ScienceDirect and Google Scholar. The search was carried out between March 2023 through September 2023.

**Result:**

The scoping search identified 17,799 records, of which 15 met the inclusion and exclusion criteria, and were fully reviewed (*N* = 15). The result shows that dehydration affects healthcare workers significantly, with 60% reporting reduced physical performance when dehydrated during shifts. Nearly half (53%) of studies document declines in cognitive pFerformance and mood. This mental strain increased with longer shift lengths, particularly among nurses on 12-h shifts and medical residents working more than 24 h.

**Conclusion:**

There is a lack of adequate studies on healthcare workers’ hydration health and its impact on performance, cognition, and mental well-being. Future research should be conducted in both cross-sectional and experimental methodologies, incorporating quantifiable measures to evaluate hydration and interventions. Countries with hot climate, such as, Saudi Arabia, should lead this research agenda, given that high temperatures could amplify the effects of inadequate hydration.

## Introduction

Healthcare workers (HCWs) perform life-saving tasks in fast-paced settings and are required to make focused and responsible executive decisions (1) in working with patients in need. A recognized aspect of work performance is adequate hydration (2). Unfortunately, HCWs are at risk of inadequate hydration for several reasons ranging from lack of resources to poor lifestyle habits (3). Inadequate hydration can have an impact on poor decision making, and there can be an increased risk of error in delivering treatment to patients (4). That is why it is important that the HCWs maintain sufficient hydration levels to preserve their ability to deliver safe service to patients and provide optimum medical performance (5). In this scoping review medical performance is used to refer to the quantifiable assessment of how effectively healthcare providers execute medical actions, behaviors, and clinical processes to achieve desired patient health outcomes.

World Health Organization recommends adequate hydration to achieve optimal performance at the workplace (6). Moreover, sufficient hydration is responsible for optimized cognition, digestion, circulation—energy levels, electrolytes—cardiac function; metabolism, thermoregulation, renal function; and for reducing renal calculi formation, headaches, and bone pathologies (7). El-Sharkawy et al. (5) add that all biological cells require water to function, making sufficient fluid intake imperative for the mind and body to perform optimally.

HCWs work in high-intensity environments. For example, they work in demanding work areas such as emergency departments, intensive care units, operating theatres, and acute care ward. Work settings of this nature come with heavy workloads, emotional demands, and time-sensitive, high-stakes clinical decisions. HCWs have little control over their work settings and conditions, and this means they may experience burnout because dehydration (8). Booker et al. (9) point out that HCWs may be at risk of insufficient water intake compared to other profession as they make up the largest proportion of shift workers. Indeed, shift work is known to be associated with circadian disruptions affecting thirst, satiety, sleep—all of which are essential for cognitive and physical performance (10).

Insufficient water consumption may also be because of lack of resources, accessibility, time or lack of awareness and responsibility (11). A study by Dutt et al. (12) highlights that nearly 40% of HCWs cite poor infrastructure within healthcare settings as a contributing factor their poor hydration. This is challenging for HCWs, for instance, nurses, who are known to walk between six to eight kilometers per 12-h shift without proper access to hydration (13). Forty percent of doctors say they feel they are unable to take a food or drink break during their shift due to the fast-paced nature of the job (14), which can prompt loss of fluids from the skin. Sweating results in a loss of 2.0 L/h to 6 L/d in high temperatures and during activity (15). HCWs are most often walking and very active, as a result they are at risk of inadequate fluid input/output. In addition, behavioral factors, such as HCWs refraining themselves from drinking to avoid bathroom breaks (16) and other associated habits stemming from lack of conscious awareness also contribute to the reduced total daily water intake. Research indicates that avoidance of bathroom breaks can cause multiple renal issues including bladder distention and an increased likelihood of urinary tract infections which can contribute to unnecessary sick leave (17).

Hydration status awareness is importance to healthcare workers because it directly influences their overall well-being, performance, and patient care. Research has shown that even mild dehydration can lead to cognitive impairments, decreased alertness, and reduced physical abilities (18). Dehydration can negatively affect HCWs cognitive functions, including memory, attention, and decision-making abilities, which are crucial for accurate and timely patient care (19). Proper hydration, on the other hand, has been associated with improved cognitive performance, mood, and job satisfaction (20). Moreover, maintaining optimal hydration status can enhance physical endurance for healthcare workers who often engage in physically demanding tasks (15). Thus, the promotion of hydration status awareness in healthcare settings can support the well-being and performance of their workers, ultimately leading to better patient outcomes.

The body's water homeostasis is maintained by neural pathways that coordinate feedback responses to neurohormones produced for the adjustment of diuresis, natriuresis and blood pressure (angiotensin mineralocorticoids, vasopressin, atrial natriuretic factor). When a person becomes hypoosmotic, the anterior hypothalamus and preoptic area show enhanced firing, which decreases when water is administered (15). Human cells require water to operate, making sufficient hydration imperative for the mind and body to perform optimally.

Imbalanced water homeostasis can lead to cognitive impairment (21). Approximately 60% of the human body is made up of water (10) and water accounts for 75% of brain mass (22). This means water is not only critical for daily work performance but for life itself. In the U.S.A, The National Academies of Sciences, Engineering, and Medicine determined that an adequate daily fluid intake is about 3.7 L for men and 2.7 L for women (23). Mild dehydration impairs several cognitive functions, including concentration and alertness. Cvirn et al. (24) found that 2.8% dehydration impairs perception, short-term memory, arithmetic ability, and psychomotor ability—all reversed with water administration (15). As little as two percent dehydration can also impair visuomotor and physical abilities (25).

The Medical Protection Society (14) indicated that National Health Society (NHS) doctors did not feel able to have a food or drink break during the working day. Moreover, it has also been reported in a survey that nearly 60% of nurses had not taken adequate breaks in their shift (26). It has been shown in a prospective cohort study set in America that 45% of HCWs were dehydrated and this had an impact on cognitive skills (5).

Abdulsalam et al. (27) conducted a cross sectional on hydration in the UAE. The participants were medical students. The study found that there is a lack of knowledge in the Gulf society about hydration markers and the importance of water because only 51% of the participants correctly recognized its importance. Moreover, 59.2% of the students admitted to drinking 2 or less bottles a day (less than 1 L a day). A further 13.9% of participants would consume more than 4 bottles (2 L a day); and therefore, most subjects remained perpetually dehydrated. However, a study conducted in Saudi Arabia found that 74% of surveyed public participants had knowledge about the importance of hydration and consumed sufficient water (28). Another study found that 59% of the participants met the minimum daily requirement of 3 L a day. Indeed, the authors recommend new innovative approaches to enhance healthy drinking with a promotion of increasing communication with the different healthcare providers (29). However, in Saudi Arabia, no specific studies on HCWs’ hydration and performance have been identified yet.

There is a pressing need for studies exploring healthcare worker hydration owing to the significant implications it holds for their well-being and job performance. Research has shown that healthcare workers often experience high levels of physical exertion, long work hours, and high-stress environments, leading to increased fluid loss and dehydration (30, 31). Dehydration can have detrimental effects on cognitive function, including impairments in memory, attention, and decision-making abilities, which are critical for accurate and timely patient care (32). Understanding the hydration needs and challenges faced by healthcare workers can provide insights into the development of effective interventions and guidelines to optimize their hydration status, ultimately improving their overall well-being and performance. By conducting studies on healthcare worker hydration, we can ensure that their hydration needs are adequately addressed, leading to better patient outcomes and a healthier workforce.

This scoping review maps the literature that examines the fluid hydration status of HCWs and the impact it has on medical performance. The search was done without specifying the geographical location given the very limited number of publications on this topic.

This review also identifies any gaps in current literature to determine what is known about HCWs hydration, and what the outcomes are and what can be done to improve hydration status to help create better outcomes for patients and HCWs. Thus, this review's main research question is: What is known in current literature on the fluid hydration status of healthcare workers and does the fluid hydration status impact their performance?

## Methods

A scoping review is the most appropriate choice for this study because the topic is broad and there may be limited research available. This review utilizes Arksey and O'Malley's (33) five-stage methodological framework: 1) identification of research question, 2) Identifying relevant studies, 3) Selection of studies, 4) Charting the data, 5) Collate, summarize, and report the result. A scoping review combines research evidence through a careful and clear process that helps to comprehensively pinpoint and dissect all the relevant literature pertaining to a research question (34). During the study selection of the current study, two reviewers independently went through the title, abstracts, and full texts of extracted articles. Any disagreements between the reviewers were resolved through discussion. However, where necessary the third reviewer was consulted to resolve the differences and reach consensus.

### Identification of relevant studies

The search was conducted between March 2023 through September 2023 using Pubmed/PubMed Central, ScienceDirect and Google Scholar using PICO method. PICO method helps in the inclusion/exclusion process and in reducing bias to ensure that relevant components of the question are adequately defined (35). It also helps researchers to conduct focused literature searches and is endorsed by the Cochrane Handbook for Systematic Reviews of Interventions (36). Articles were included based on inclusion/exclusion criteria. The articles had to be peer-reviewed, had focused on healthcare hydration status and performance between 2005 and 2023, and published in English language only. Records were excluded if they were not published in English, did not focus on healthcare hydration and performance, and were published outside the stated time frame. Gray literature including dissertations, thesis, reviews, reports, and conference papers were excluded.

### Search word strategy plan

This review employed the following pattern to search for article about HCWs hydration status and performance. Hydration status OR hydration OR fluid intake OR fluid status OR Hydration fluid status AND Healthcare professionals OR healthcare workers OR nurses OR doctors OR healthcare OR shift work AND performance OR impact OR outcome. Table 1 in the appendices shows the initial phase of article searches and review yielded.

### Study article screening process

The articles were screened using PRISMA guidelines (37), which structures the screening process into organizable chunks and categories. The literature review process confirmed data points and areas of relevance where the following were noted: study title, authors name, year, type of study, location, study population, aims, findings, method limitations and recommendations for future research and policy. Moreover, thematic analysis (38), was used to identify, analyze, and report themes within data. First, key words were utilized in search engines to generate all types of relevant publications pertaining to hydration in HCWs. After that, a data table was created using the following codes: citation, study design, sample size, sample population, journal, aims, key findings, limitations to methods, hydration measurement tools, performance evaluation tools, and additional key findings. Next, themes were searched using data from the table. The last step was to review the themes, for example, prevalence of dehydration in HCWs during their shifts, hydration status in HCWs, examine if gender or age differences or other demographic differences and how dehydration affects performance and cognition. Table 2 (appendices) presents the PRISMA-ScR checklist based on Tricco et al. (37) to show transparency, completeness and reporting of the current scoping reviews.

## Results & discussion

**Figure 1:**
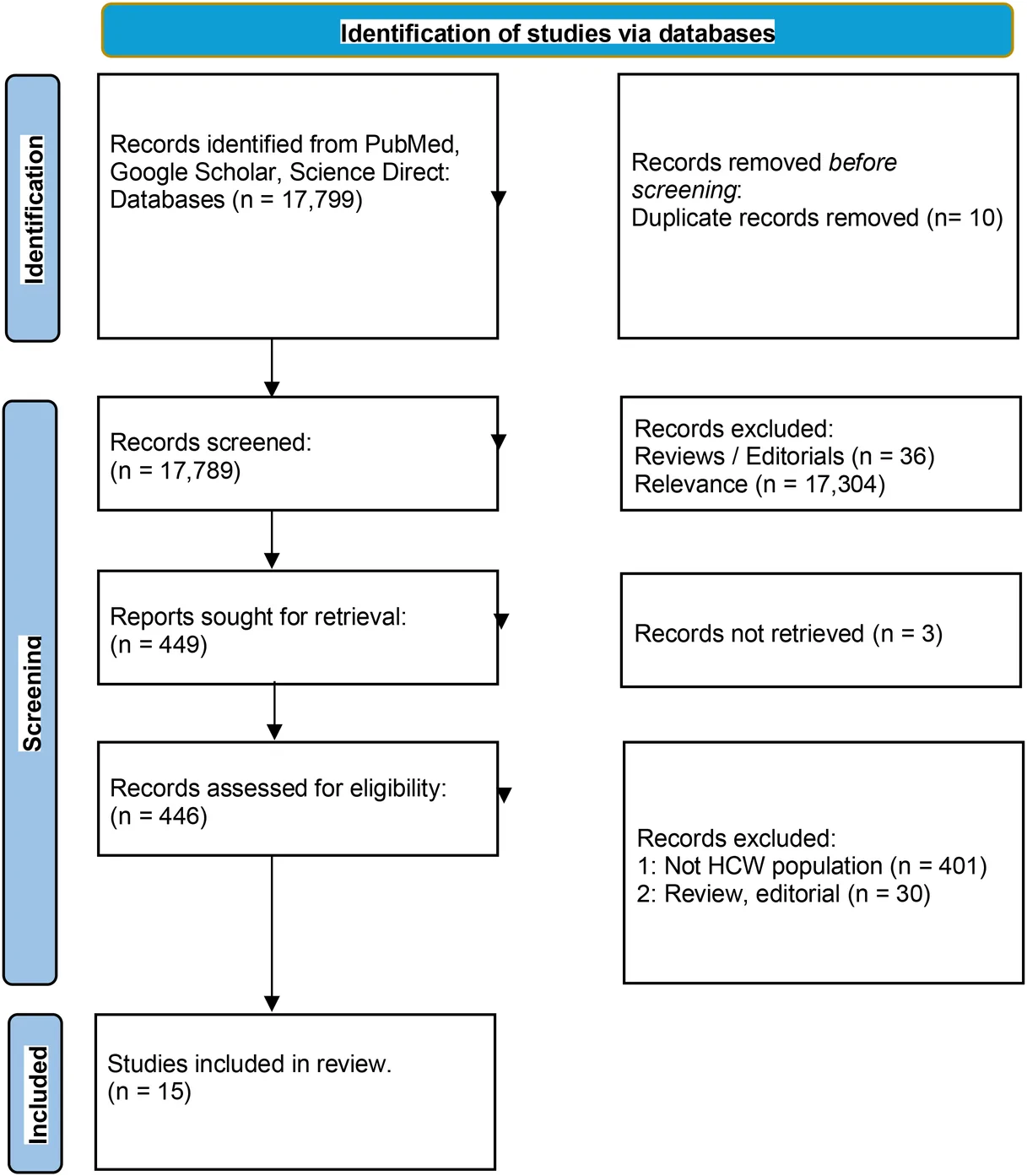
The PRISMA flow chart (39) results are summarized.

As shown in Figure 1, from 17,799 records originally identified from the searches, a total of 15 articles met the final inclusion criteria for the review. The list of articles is shown in Table 3.

**Table 3:** Summarized results of the scoping review.

No.	Authors	Title	Year	Duration/months	Country/setting	Primary Aim/study domain	Study design	Impact/symptoms	Predictor dehydration
1	Alomar et al. (4)	Decreased hydration status of emergency department physicians and nurses by the end of their shift	2013	6 months	Saudi Arabia	Healthcare worker hydration	Cross sectional survey	Poor concentration/mood and mood, sweating, feeling thirsty, reduced physical performance	Young by age, Starting shift in dehydrated state, Day shift, Limited fluid intake, Limited fluid intake—intentional
2	Blake et al. (40)	Mitigating the Psychological Impact of COVID-19 on Healthcare Workers: A Digital Learning Package	2020	12 months	United Kingdom	Healthcare worker wellbeing	Comparative descriptive study	poor concentration/mood, reduced physical performance	Limited fluid intake—time constraint
3	Sunardi et al. (41)	Water and beverages intake among workers amid the Covid19 pandemic in Indonesia	2022	2 weeks	Indonesia	Healthcare worker hydration	Cross sectional survey	Reduced urine output, reduced physical performance	Limited fluid intake—time constraint, Overheating (PPE or ambient temperature) causing perspiration
4	Davey et al. (43)	Heat stress and PPE during COVID-19: impact on healthcare workers’ performance, safety and wellbeing in NHS settings	2021	4 months	United Kingdom	Effects of PPE	Cross sectional survey	Reduced physical performance, exhaustion/fatigue	Overheating (PPE or ambient temperature) causing perspiration
5	El-Sharkawy et al. (5)	Hydration amongst nurses and doctors on-call (the HANDS-on prospective cohort study)	2016	13 months	United Kingdom	Healthcare worker hydration	Cohort	Reduced urine, poor concentration/mood, dizziness	Starting shift in dehydrated state, Limited fluid intake—time constraint, Overheating (PPE or ambient temperature) causing perspiration, Lack of hydration importance awareness
6	Grelot et al. (44)	Moderate Thermal Strain in Healthcare Workers Wearing Personal Protective Equipment During Treatment and Care Activities in the Context of the 2014 Ebola Virus Disease Outbreak	2016	3 months	Guinea	Effects of PPE	Experimental	Poor concentration/mood, headache, reduced urine output	Overheating (PPE or ambient temperature) causing perspiration
7	Hwang et al. (45)	Total Fasting and Dehydration in the Operating Room: How Can Surgeons Survive and Thrive?	2021	7 days	USA	Healthcare worker hydration	Cross sectional survey	Headache, reduced physical performance, poor concentration/mood	Limited fluid intake—intentional, Overheating (PPE or ambient temperature) causing perspiration
8	Lee et al. (46)	Heat Stress and Thermal Perception amongst Healthcare Workers during the COVID-19 Pandemic in India and Singapore	2020	4 months	India and Singapore	Effects of PPE	Cross sectional survey	Sweating, exhaustion, fatigue. Reduced urine output.	Limited fluid intake—time constraint, Limited fluid intake—intentional, Overheating (PPE or ambient temperature) causing perspiration, Lack of hydration importance awareness
9	Makowski et al. (47)	Performance Nutrition for Physician Trainees Working Overnight Shifts: A Randomized Controlled Trial	2022	7 months	USA	Healthcare worker wellbeing	Controlled, block randomized crossover trial	exhaustion, poor concentration/mood, reduced physical performance	Limited fluid intake—time constrain, Starting shift in dehydrated state
10	Messeri et al. (48)	A Web Survey to Evaluate the Thermal Stress Associated with Personal Protective Equipment among Healthcare Workers during the COVID-19 Pandemic in Italy	2021	5 months	Italy	Effects of PPE	Cross sectional survey	Sweating/perspiration, fatigue, poor concentration/mood	Limited fluid intake—intentional
11	Ong et al. (49)	Headaches Associated with Personal Protective Equipment—A Cross-Sectional Study Among Frontline Healthcare Workers During COVID-19	2020	1 month	Singapore	Effects of PPE	Cross sectional survey	Headache, feeling thirsty/dry mouth	Overheating (PPE or ambient temperature) causing perspiration
12	Palejwala et al. (50)	Effects of a hot ambient operating theatre on manual dexterity, psychological and physiological parameters in staff during a simulated burn surgery	2019	2 months	Australia	Healthcare worker wellbeing	Experimental	Reduced urine output, reduced physical performance, loss of 1%–2% of body weight	Overheating (PPE or ambient temperature) causing perspiration
13	Pierce et al. (51)	Delaying voiding, limiting fluids, urinary symptoms, and work productivity: A survey of female nurses and midwives	2019	5 months	Australia	Healthcare worker hydration	Cross sectional survey	Reduced physical performance, poor concentration/mood, headache	Limited fluid intake—time constraint, Limited fluid intake—intentional
14	Rojo-Rojo et al. (52)	Risk of Dehydration Due to Sweating While Wearing Personal 2 Protective Equipment in COVID-19 Clinical Care: A Pilot Study	2022	2 months	Spain	Effect of temperature	Experimental	Feeling thirsty, dry mouth, loss of 1%–2% of body weight,	Limited fluid intake—time constraint, Limited fluid intake—intentional, Overheating (PPE or ambient temperature) causing perspiration
15	Swaminathan et al. (53)	Impact of enhanced personal protective equipment on the physical and mental well-being of healthcare workers during COVID-19	2022	1 months	United Kingdom	Effects of PPE	Cross sectional survey	Headache, exhaustion, fatigue, reduced physical performance, sweating	Overheating (PPE or ambient temperature) causing perspiration

### Analysis of inclusion articles

Although a limited number of articles met the criteria of healthcare workers and hydration outcomes, an analysis of the available inclusion articles provides perspective on trends and patterns that speak to the justification of this report as well as highlighting the need for imminent studies in the area, both in Saudi Arabia and globally. Given the limitations on existing publications on the topic, this review did not apply many filters towards acquiring the review articles, namely, the range of years was set to be from 2005 to 2023 and articles had to be from peer-reviewed, reputable journals published in either PubMed, ScienceDirect or Google Scholar. However, Google Scholar yielded many records. This result was screened and restricted to the first 150 by relevance. After that title, abstracts and full articles were scrutinized against pre-defined inclusion/exclusion criteria resulting in 6 articles for final review. This decision aligns with scoping review methodology, where the aim is to map key literature rather than conduct an exhaustive systematic search (33). The results of the scoping review are summarized in Table 3.

The findings as indicated in Table 3 illustrate that the rise in studies looking into healthcare worker wellness by way of nutrition and hydration peaked during COVID19 studies, where it reached six of 15 from a low of 1–2 articles per year. Notably, of the 15 studies, eight were COVID19—specific studies. The COVID-19 pandemic significantly accelerated the publication of studies on healthcare workers' well-being, highlighting the urgent need to address this critical aspect. Numerous research articles emerged in response to the unprecedented challenges faced by healthcare professionals during the pandemic. These studies shed light on the profound impact of the crisis on healthcare workers' mental health, job satisfaction, burnout rates, and overall well-being (54). For instance, research has shown that the increased workload, fear of infection, and lack of personal protective equipment have contributed to heightened stress levels among healthcare workers (55). Moreover, studies have explored the effectiveness of various interventions, such as mental health support programs and resilience training, in mitigating the negative effects on well-being (56). The surge in published studies during the pandemic indicates the recognition of healthcare workers' well-being as a crucial determinant of the overall healthcare system's resilience and effectiveness. These studies provide valuable insights into the development of evidence-based interventions to support and protect the well-being of healthcare professionals during times of crisis.

The duration of study of the reviewed articles ranged from seven days to around 13 months (summarized in Table 3). The average duration of study was two months of study at 20% of articles, followed by five months, six months, and 12 months at 13.3% of articles each respectively. This shows that more studies were done for longer periods of time rather than short-term studies. This is a good indication that the inclusion articles collected data for longer times to yield more representative result.

The findings from the current study also indicate that most hydration investigations in healthcare workers was done in the United Kingdom (4 of 15 studies), followed by the United States of America (*n* = 2) and Indonesia (*n* = 2). Other countries, including Saudi Arabia, has only one study. This highlights the need for more global studies on the subject matter and shows that an international interest is persistent in the topic of healthcare workers hydration and outcomes.

The articles that were included in the review had varied primary aims. Not all studies were directly focused on HCWs hydration, but rather the ones included had a focus on healthcare workers and measured hydration and outcome to some extent.

The current research shows that studies exploring the effects of PPE on healthcare workers (*n* = 7) was most of the inclusion studies, while direct studies on hydration in healthcare workers followed closely behind (*n* = 5). Studies on healthcare worker well-being (*n* = 2) and the effects of temperatures on healthcare workers performance (*n* = 1) were the least common objectives. This highlights that there are limited studies on the well-being of healthcare workers. Medical settings are encouraged to implement interventions that intervene at both the environmental and personal level to help facilitate behavior change among health care providers (57).

As varied as the objectives were, study designs were also varied. However, one study design dominated the landscape.

Moreover, as depicts in Table 3 nine of fifteen (60%) of studies were survey based, as cross-sectional studies. Experimental studies followed behind with three of fifteen (20%), and the remaining study designs were cohort (*n* = 1), comparative (*n* = 1) or randomized crossover trials (*n* = 1). This illustrates the studies into healthcare worker hydration outcomes are largely conducted by surveys, making the results subjective. This prompts the recommendation that more studies that are experimental be done to ascertain objective measures of hydration status and the outcomes of intervention strategies. Every one of the studies were conducted in hospitals. Of the fifteen articles, fourteen studies included a sample population of physicians, one study was only conducted on nurses; fourteen studies included a nurse sample population, and a single study was conducted only on physicians. Two studies included healthcare administrators, one study included physiotherapists and one study included paramedics; one study was done strictly in the operating room, two studies focused on the emergency department and ambulatory services. This depicts a limited sample population being examined in the healthcare worker field and prompts the recommendation that future studies include all types of workers in hospital and other healthcare settings, to confirm a more representative image of the state of hydration amongst healthcare workers.

Not all the studies included had distinct measurements of hydration. However, four of the studies included measurements of hydration with the following pattern: four measured body weight and calculate BMI, two studies employed a urinalysis to examine ketones and specific gravity as a measure of hydration, three studies recorded core body temperatures, one record measured fluid loss, one research used a thermal body camera to record heated areas of the face and body, one study utilized a cognitive performance test to evaluate the effect of dehydration, and lastly one study tested subjects on performance impact of dehydration with tests on psychomotor vigilance, sensory-motor speed, working memory, risk decision making.

Moreover, of the nine cross-sectional survey-based studies: six asked subjects about symptom outcomes from being dehydrated (nausea, dizziness, headaches, exhaustion, fainting, thermal sensation/perspiration), and nine asked about levels of thirst.

To that end, it is worthy to review both predictors of dehydration and outcomes of dehydration as evaluated by the inclusion articles. While this report intended to note the effects of poor hydration on performance, the limited number of studies yielded did not make this possible. Nevertheless, from the studies an analysis of both contributing factors causing dehydration as well as various sequelae of dehydration in HCWs is reviewed in the figures and tables below.

Table 3 shows that 67% of the inclusion articles (*n* = 10) associated PPE overheating and subsequent perspiration as a predictor of reduced hydration in healthcare workers. A limited number of breaks and/or lack of access to break rooms was the next commonest predictor for HCWs not consuming sufficient fluids (*n* = 8). Interestingly, 40% of studies (*n* = 6) noted intentional fluid restriction, a technique employed by healthcare workers to not consume fluids so that they do not have the need for bathroom breaks during the shift. Some attributed this to poor facilities, others to the lack of desire to don and doff PPE for each bathroom visit, and many attributed the intentional restriction to a lack of time during the shift, especially in emergency departments and operating rooms, where it is not optimal to disrupt essential work with unwelcomed bathroom urges. Three studies noted that many healthcare workers start their shift in a dehydrated state, making the lack of fluid consumption worse during the shifts. Two studies highlighted that when asked, HCWs reported having a limited awareness about the importance of hydration during a shift and the potential outcomes of sustained dehydration. The figure below visually summarizes the dehydration outcomes findings.

The impact of dehydration among healthcare workers is also noted in all the 15 studies (see Table 3). It is worth noting that not all studies inquired into all the mentioned symptoms, however, based on the analysis of what was mentioned the figure above depicts that 9 of 15 studies (60%) reported reduced physical performance on the job during dehydrated states on shift. This would be a highly significant finding given that healthcare workers by and large spend a huge amount of time on their feet, tending to patients, and transferring samples, information, and resources. A reduction in physical performance warrants a need for hydration interventions in the healthcare setting. Physicians often work more than 16 h, which intensifies fatigue and has been shown to increase the number of preventable adverse events leading to patient death by 300% (57). Closely following is eight of 15 (53%) of studies noting a reduction in cognitive performance and mood. Again, the ability to concentrate, focus and make executive decisions is imperative to making life-saving decisions in the healthcare setting. Any reduction in this state puts patients' lives at risk. Caring for patients is mentally stressful and this phenomenon increases as shift length increases, where healthcare workers tend to work longer hours than the general population, with an average of 12-h shifts for nurses, while medical residents shift last longer than 24 h (57, 58). Furthermore, a low mood or affect will be felt in the patient experience and affects others in the working environment. Both factors are essential to optimal working environments and prompt the need for more hydration studies and more hydration interventions. The risk of death from medical error in the UK is almost one per day (1 in 300), where the highest risk is noted to be in the operating rooms, where the tiniest error can have exponentially severe and detrimental impacts on patients (59).

### Healthcare strategies & policy recommendations

The studies that were included in the scoping review show several strategic recommendations to address the issue of HCW dehydration. Firstly, improving access to bathroom facilities particularly because many dehydrated HCWs purposefully dehydrate due to lack of access to bathrooms. Furthermore, enforcing break times and making adequate well-being spaces for breaks and refreshments, especially for emergency department and ambulatory staff. Other authors have strongly recommended that healthcare workers drink regularly and stop for short breaks every 2–3 h (59). Another important recommendation is to increase access to drinking water, i.e., water dispensers in all working areas, as many respondents in the reviewed studies reported not drinking due to limited access to water or refreshments.

Placing hydration and diet information posters throughout health facilities could also be considered as this may increase hydration awareness and prompt workers to take water and bathroom breaks to maintain health. Another suggestion includes placing electrolyte packs and food bars close to surgical wards and operating rooms for access by HCWs in operating rooms. Another study suggested that ambient temperature be regulated in working spaces to prevent overheating and excess perspiration. Several studies encouraged pre-shift hydration. Surprisingly, there are no apparent published studies about the importance of breakfast for HCW staff (59).

Lastly, most studies suggested that more studies be conducted to explore the prevalence of hydration status in HCWs and the impact on cognitive and physical performance, to justify hospital wide policies to bring attention to the matter.

Some existing examples of regional policies include The European Healthy Hydration Awareness Campaign (EuHHAC), which is an initiative of the European Federation of the Associations of Dietitians (EFAD) and the Hydration and Health Department of Danone Research. This program aims to increase knowledge and awareness of healthy hydration and since 2016, the EuHHAC has conducted an ongoing online survey to address gaps in hydration knowledge, a webinar providing information on the impact of hydration on health and well-being, an interactive session addressing barriers to hydration, and a tutorial summarizing hydration information, to which dietitians were actively engaged in each aspect (60).

There is a definite need to nurture a medical culture that increases awareness of the relationship between nutrition and well-being among physicians, and to prioritize self-care amongst HCWs through nutrition. For example, the Accreditation Council for Graduate Medical Education and the American Board of Pediatrics have incorporated nutrition wellness as part of the core competencies (61).

Other suggested strategies include:

1. Education and Awareness

By developing comprehensive educational programs to raise awareness about the risks of dehydration among healthcare workers, there is a chance of improved outcomes. These programs should highlight the signs and symptoms of dehydration, the importance of proper hydration, and practical strategies to maintain adequate fluid intake during shifts. Clinicians and healthcare staff are often unaware that they are inadequately hydrated, and this could affect their performance. Therefore, hospital leaders and managers should promote the importance of regular breaks for staff even when they are “too busy to stop” (59, 62). Perhaps in this case wearable devices may play a role in the future of prompting HCWs when approaching a state of dehydration.

2. Training

Integrating hydration training into healthcare worker education and training programs can empower them with the knowledge and skills to recognize and address dehydration effectively. Training should cover topics such as the effects of dehydration on performance, the importance of early intervention, and practical strategies for staying hydrated. Examples of interventions include individual motivational counseling, team goal setting exercises, physical activity sessions, and relaxation programs, all of which have been shown to result in improved employee dietary habits, body mass index, waist circumference, physical activity levels, perceived stress, and the onset of work-related disabilities (57).

3. Support Systems

Establishing support systems within healthcare facilities can play a significant role in addressing healthcare worker dehydration. This can include providing access to hydration resources, such as water coolers or hydration stations, and ensuring that healthcare workers have adequate time for breaks during their shifts.

4. Monitoring and Assessment

Implementing systems to monitor healthcare worker hydration levels can help identify at-risk individuals and address their needs promptly. This can involve regular hydration assessments or the use of wearable technology that tracks hydration status.

Improving drinking habits during working time whilst developing hydration strategies and guidelines for healthcare professionals and patients would be a cost-effective means of addressing the burden of hypohydration in developed countries (63).

### Key findings

The current study highlights the following key outcomes:

1. There is a lack of studies on healthcare workers’ hydration health.
2. There is a lack of studies on impact of healthcare worker dehydration status and impact on performance, cognition, and mental well-being.
3. COVID studies prompted a rise in HCW well-being studies, many considered hydration status (as after donning PPE hydration potential was limited and potentiated heat stress related illnesses).
4. Ample break times during shifts allow staff time to rest, rehydrate and refocus.

### Limitations

This report encountered limitations. The most prominent limitation was the extremely limited number of published articles on the topic of healthcare worker hydration status, and furthermore on the outcomes of dehydration. A second limitation is the availability of only a single study conducted in Saudi Arabia, making secondary objectives incomplete, as a comparison was not feasible due to limited information and studies. A third limitation is a lack of a prior protocol registration because it is not mandatory in scoping reviews, and this may limit methodological transparency.

## Conclusions

Dehydration among healthcare workers is a pressing issue that can affect both their well-being and the quality of patient care. By implementing healthcare policies and strategies that focus on prevention, education, and support, healthcare facilities can effectively address this challenge. This report highlighted the very limited number of studies conducted on the topic, which has serious implications on the quality of care distributed to patients under the care of HCWs. The review concludes that it is imperative that future studies be conducted in both cross-sectional and experimental methodologies, incorporating quantifiable measures to evaluate hydration and the interventions to explore the impacts on HCWs. Additionally, the studies should be conducted at a worldwide level. Moreover, Saudi Arabia is encouraged to lead this venture, especially given the high temperature climate that would further amplify the effects of reduced hydration in healthcare workers. It is crucial for healthcare organizations to prioritize the hydration needs of their workers to ensure a healthy and productive workforce, ultimately benefiting both healthcare workers and the patients they serve (57–61).

## Data Availability

The original contributions presented in the study are included in the article/Supplementary Material, further inquiries can be directed to the corresponding author.

## Appendix

**Table 1:** Shows the initial phase of article searches, and reviews yielded.

Source	Search words	Total Articles	Duplicates/Reviews	Articles After Screening Title & Abstract & URL
PubMed	healthcare workers AND hydration AND performance	93		4
PubMed	fluid intake AND healthcare workers AND impact	18		1
PubMed	dehydration AND healthcare workers AND performance	76	Duplicates—4 Reviews—14	2
Google Scholar	Healthcare workers AND hydration AND performance (2005 to 2023)	17,600	Duplicates—6 Reviews—15	6
Science Direct	Healthcare workers AND hydration status AND performance	12	Reviews—7	2
TOTALS		17,799		15

**Table 2:** (PRISMA-ScR) checklist.

Section	Item	PRISMA-ScR item	Reported on section
Title			
Title	1	Identify the report as a scoping review.	Title
Abstract			
Structured summary	2	Provide a structured summary	Abstract
Introduction			
Rationale	3	Describe the rationale for the review	Introduction
Objectives	4	Provide an explicit statement of the questions and objectives.	Introduction
Methods			
Protocol and registration	5	Indicate whether a review protocol exists	Not applicable
Eligibility criteria	6	Specify characteristics of the sources of evidence used as eligibility criteria	Identification of relevant studies
Information sources	7	Describe all information sources in the search	Identification of relevant studies
Search	8	Present the full electronic search strategy	Search word strategy plan
Selection of sources of evidence	9	State the process for selecting sources of evidence	Study article screening process
Data charting process	10	Describe the methods of charting	Study article screening process
Data items	11	List and define all variables for which data were sought	Initial search results
Critical appraisal of individual sources of evidence	12	If done, provide a rationale for conducting a critical appraisal, the method used and how the results were applied.	Not applicable
Synthesis of results	13	Describe the methods of handling and summarizing the data that were charted.	Analysis of inclusion articles
Results			
Selection of sources of evidence	14	Give numbers of sources of evidence screened	Characteristics of included studies
Characteristics of sources of evidence	15	Present characteristics for which data were charted	Characteristics of included studies
Critical appraisal within sources of evidence	16	If done, present data on critical appraisal of included sources of evidence	Not applicable
Results of individual sources of evidence	17	Present charted data	Healthcare Workers Hydration Status and Performance
Synthesis of results	18	Summarize charting results	Healthcare Workers Hydration Status and Performance
Discussion			
Summary of evidence	19	Summarize the main results	Results and discussion
Limitations	20	Discuss the limitations of the scoping review process.	Limitations
Conclusions	21	Provide a general interpretation of the results	Conclusion
Funding			
Funding	22	Describe sources of review.	No funding
